# Facile preparation of Zn_*x*_Cd_1−*x*_S/ZnS heterostructures with enhanced photocatalytic hydrogen evolution under visible light

**DOI:** 10.1039/d1ra03195c

**Published:** 2021-06-18

**Authors:** Jing Dong, Wenjian Fang, Weiwei Xia, Qihong Lu, Xianghua Zeng

**Affiliations:** College of Chemistry and Chemical Engineering, Yangzhou University Yangzhou 225002 P. R. China; College of Electrical, Energy and Power Engineering, Yangzhou University Yangzhou 225127 P. R. China xhzeng@yzu.edu.cn; College of Physics Science and Technology & Institute of Optoelectronic Technology, Yangzhou University Yangzhou 225002 P. R. China

## Abstract

Hydrogen evolution from water using solar energy is regarded as a most promising process, thus, exploring efficient photocatalysts for water splitting is highly desirable. To avoid the rapid recombination of photogenerated electrons and holes in CdZnS semiconductors, Zn_*x*_Cd_1−*x*_S/ZnS composites were synthesized *via* a one-step hydrothermal method and then annealed at 400 °C for 60 min under argon flow. Zn_*x*_Cd_1−*x*_S/ZnS composites are composed of ZnS nanosheets decorated with Zn_*x*_Cd_1−*x*_S nanorods, and TEM and UV-vis absorption spectra confirm the formation of the heterostructure between Zn_*x*_Cd_1−*x*_S nanorods and ZnS nanosheets. Because of the well-matched band alignment, stronger optical absorption and larger carrier density, Zn_0.2_Cd_0.8_S/ZnS has the highest hydrogen production, with a photocatalytic hydrogen production rate up to 16.7 mmol g^−1^ h^−1^ under visible light irradiation. Moreover, the photocatalyst also exhibits high stability and good reusability for hydrogen production reaction. The facile and efficient approach for ZnS based heterostructures could be extended to other metal compound materials.

## Abstract 2

Schematic illustration for electron charge transfer and H_2_ evolution mechanism for the Zn_0.2_Cd_0.8_S/ZnS nanocomposites.

## Graphical abstract

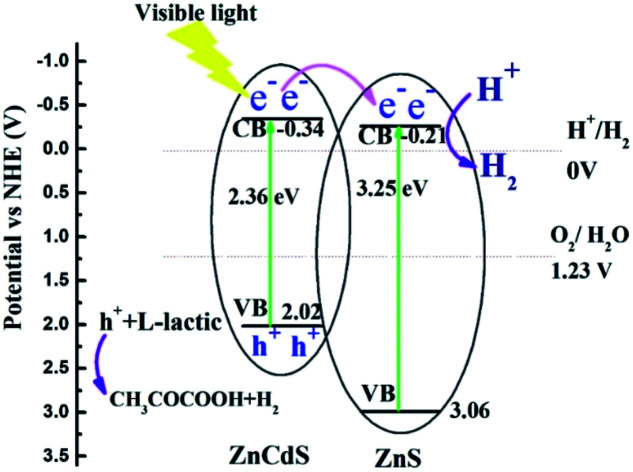

## Introduction

In consideration of the global energy crisis and environmental issues, much effort has been made to replace exhaustible fossil fuels with clean energy sources. Hydrogen, with a fuel value (∼143 kJ g^−1^) three times higher than that of gasoline and only non-polluted water as a by-product, has been regarded as one of the most important green energy carriers to replace fossil fuels. Among various strategies for hydrogen evolution, photocatalytic hydrogen evolution is regarded as the most promising way from water splitting using solar energy, because of the direct utilization of solar energy to achieve H_2_ generation from water splitting, as well as its much simpler and more economically competitive systems.^1^

Ternary ZnCdS nanomaterials were regarded as a promising substitute for noble-metal co-catalysts in photocatalytic reactions because of their tunable band gap energy and lattice constant between 0.331 (〈100〉 of ZnS) and 0.359 nm (<〈100〉 of CdS) in comparison with ZnS or CdS materials.^2–6^ Recently some studies on ZnCdS- related photocatalytic hydrogen reactions have been reported. Such as, Jin *et al.*^7^ reported that ZnCdS catalysts with a (Zn + Cd) : S molar ratio of 1 : 3.5 has much higher photocatalytic activity and exhibited an excellent stability after six cycles. And the H_2_ production of (CdS–ZnS)–TiO_2_ supported photocatalytic system was studied by Tambwekar *et al.*^8^ The hydrogen evolution rate of ZnCdS–CdS heterostructure was obtained from 25.46 to 72.82 and 52.82 μmol h^−1^, and the value increased to 192.28 μmol h^−1^ after the adoption of VS_2_ to the heterostructure surface.^9^ GQDs/ZnCdS/PdS exhibited a H_2_ evolution rate of 517 μmol h^−1^, which is 15, 7 and 1.7 times higher than that of pure ZnCdS, GQDs/ZnCdS, and ZnCdS/PdS, respectively.^10^ α-Fe_2_O_3_/Zn_0.4_Cd_0.6_S heterostructure has visible light photocatalytic H_2_ production of 536.8 μmol h^−1^.^11^ The ZnCdS–CH solid solution without noble metal loading achieved a superior photocatalytic H_2_ activity rate of 0.971 mmol h^−1^ under visible light irradiation (*λ* ≥ 420 nm), which exceeds those of coprecipitated Zn_0.5_Cd_0.5_S samples by more than 13 times.^12^ The hydrogen production rate of Fe_1−*x*_Pt_*x*_-ZnCdS NPs had a significant enhancement over the pure ZnCdS (740 μmol g^−1^ h^−1^). The hydrogen production activity of ZnCdS–NiCoP composite catalyst was improved greatly, which reached 5.2 times that of pure ZnCdS.^13^ The highest hydrogen production rate of 2.265 mmol g^−1^ h^−1^ was achieved by the 0.5 wt% Fe_0.3_Pt_0.7_–ZnCdS nanocomposites, which was even better than that of 0.5 wt% Pt–ZnCdS (1.626 mmol g^−1^ h^−1^) under the same condition.^14^

The photocatalytic H_2_ evolution rate of Zn_0.3_Cd_0.7_S nanorods was improved from 517.4 to 3310.1 μmol g^−1^ h^−1^ by loading a suitable amount of Ni_3_C NPs as co-catalyst under visible light irradiation.^15^ Hollow Zn_0.6_Cd_0.4_S cage material exhibited the highest hydrogen production rate of 5.68 mmol h^−1^ g^−1^ under cocatalyst-free and visible-light irradiation conditions.^16^ The addition of NiB greatly improved the photocatalytic performance of CdZnS, and the hydrogen production of NiB/CdZnS catalysts reached 8.137 mmol g^−1^ h^−1^, which is 17 times that of pure CdZnS.^17^ A typical “type II” band alignment forms at Zn_0.5_Cd_0.5_S hybrided with carbon materials lead to the improved H_2_ generation rate of 10.8 mmol g^−1^ h^−1^, exceeding the Zn_0.5_Cd_0.5_S nanosheets (6.4 mmol g^−1^ h^−1^) and QDs (3.4 mmol g^−1^ h^−1^).^18^ A controlled ZnCdS QDs developed by a simple ZIF-8 templating method exhibited significantly enhanced photocatalysis performance with a H_2_ production rate of 3.70 mmol h^−1^ g^−1^ (Zn_0.5_Cd_0.5_S QDs),^19^ and the highest hydrogen production rate of Zn_0.5_Cd_0.5_S/dodecahedron ZIF-67 composite reached 23.264 mmol g^−1^ h^−1^ under visible light irradiation,^20^ which was explained as the matched valence band position for ZIF-67 and ZCS and the exposure of rich active sites. The WO_3_/ZnCdS compound catalysts showed the hydrogen production activity of the 35 wt% WO_3_/ZnCdS to 98.68 μmol mg^−1^, about 9.6 times that of pure ZnCdS (10.28 μmol mg^−1^).^21^

Although many efforts have been done to develop efficient photocatalytic systems and photocatalysts by using ZnCdS materials and their composites, it is still an urgent task to develop low-cost co-catalysts for advancing photocatalytic H_2_ production. In this paper, simple hydrothermal and annealing reaction methods were developed for preparation of pure ZnS nanosheets, CdS nanorods and Zn_*x*_Cd_1−*x*_S/ZnS composites. The photocatalytic hydrogen production rate of Zn_0.2_Cd_0.8_S/ZnS (#3) up to 16.7 mmol g^−1^ h^−1^ has been obtained under visible light irradiation with a good stability. This value is larger than the reported pure Zn_*x*_Cd_1−*x*_S/ZnS composites. Furthermore, the preparation method is simple. The outstanding photocatalytic performance benefited from the efficient charge transfer between ZnCdS nanorods and ZnS nanosheets in the Zn_*x*_Cd_1−*x*_S/ZnS heterojunctions, as well as more active sites on the surface of nanosheets. This study will be helpful to obtain catalysts for photocatalytic H_2_ production based on low-priced metal composite materials.

## Experimental section

### Materials and chemicals

All chemicals were analytical-grade and used without further purification. Zinc acetate [Zn(OAc)_2_·2H_2_O], cadmium acetate [Cd(OAc)_2_·2H_2_O], thiourea [(NH_2_)_2_CS] and ethanol were purchased from Sinopharm Chemical Reagent Co., Ltd (Shanghai China). Ethylenediamine was purchased from Aladdin Chemical Reagent Co., Ltd (Shanghai).

### Sample preparation

ZnS nanosheets, CdS nanorods and Zn_*x*_Cd_1−*x*_S/ZnS heterostructure were synthesized according to the following methods. ZnS nanosheets were prepared according to the previous reports with some modifications.^22^ Typically, 60 mM Zn(OAc)_2_·2H_2_O was dissolved in 6 mL deionized water under constant magnetic stirring for approximately 10 min, then, 24 mL ethylenediamine was added to the solution. After the mixed solution was stirred continuously and cooled to room temperature, 0.18 M (NH_2_)_2_CS was added into the above solution. After stirring for another 30 min, the mixture was transferred into a 50 mL Teflon-lined stainless steel autoclave and keeping sealed under 160 °C for 6 h. The product was collected and washed with water and ethanol for several times and dried at 60 °C for 24 h. Furthermore, the precursor was annealed in the quartz tube furnace at 400 °C for 60 min under argon (Ar) flow. After heat treatment, ZnS nanosheets were achieved, labeled as sample #1. CdS nanorods were prepared with the same procedure, except that 60 mM Cd(OAc)_2_·2H_2_O was used as cationic precursor (so called as sample #4). For Zn_*x*_Cd_1−*x*_S/ZnS heterostructures, 48 mM Zn(OAc)_2_·2H_2_O and 12 mM Cd(OAc)_2_·2H_2_O were used as cationic precursor, labeled as sample #2; 36 mM Zn(OAc)_2_·2H_2_O and 24 mM Cd(OAc)_2_·2H_2_O were used as cationic precursor, named as sample #3.

### Characterization

The crystal structures of the samples were analyzed by powder XRD (D8 Advance, Bruker-AXS) using Cu Kα (*λ* = 0.154056 nm), and their patterns were collected in the 2*θ* range from 10° to 80° using a continuous scanning method at a scanning speed of 2° (2*θ*) min^−1^. The morphology and microstructure of the as-synthesized samples were characterized using Hitachi S-4800 field emission SEM and Tecnai G2 F30 field emission TEM, operated at an accelerating voltage of 300 kV, the composition of the products was measured by energy dispersive X-ray spectroscopy (EDX). Brunauer–Emmett–Teller (BET) surface area measurements were performed with N_2_ adsorption at 77 K on a BSD-PS (M) instrument. Before analyses, the samples were degassed at 80 °C. Absorption measurements were carried out using a UV-vis-NIR spectrophotometer (UV-vis, Cary-5000, Varian). Photoluminescence (PL) measurements were performed using a Britain Renishaw InVia spectrophotometer, with a 325 nm line of a He–Cd laser as the excitation light source in a closed-cycle He cryostat. In addition, the chemical states of Zn, Cd and S elements were analyzed by X-ray photoelectron spectroscopy (XPS, Thermo ESCALAB250Xi) which was equipped with a standard monochromatic Al-Kα source (*hν* = 1486.6 eV), the binding energies were referred to C 1s peak (284.8 eV).

The PEC analysis was carried out on Zahner CIMPS electrochemical workstation (Germany) in a quartz cell, using a three-electrode cell with a Pt wire as the counter electrode, Ag/AgCl electrode as the reference electrode and FTO glass covered with photocatalyst as the working electrode. A 300 W Xe lamp (CEL-HXF 300, Beijing Au-light, China) was employed as an incident light source to study the PEC response of the samples, and Na_2_SO_4_ (0.2 M) solution was used as the electrolyte.

### Photocatalytic H_2_ production

**Fig. 1:**
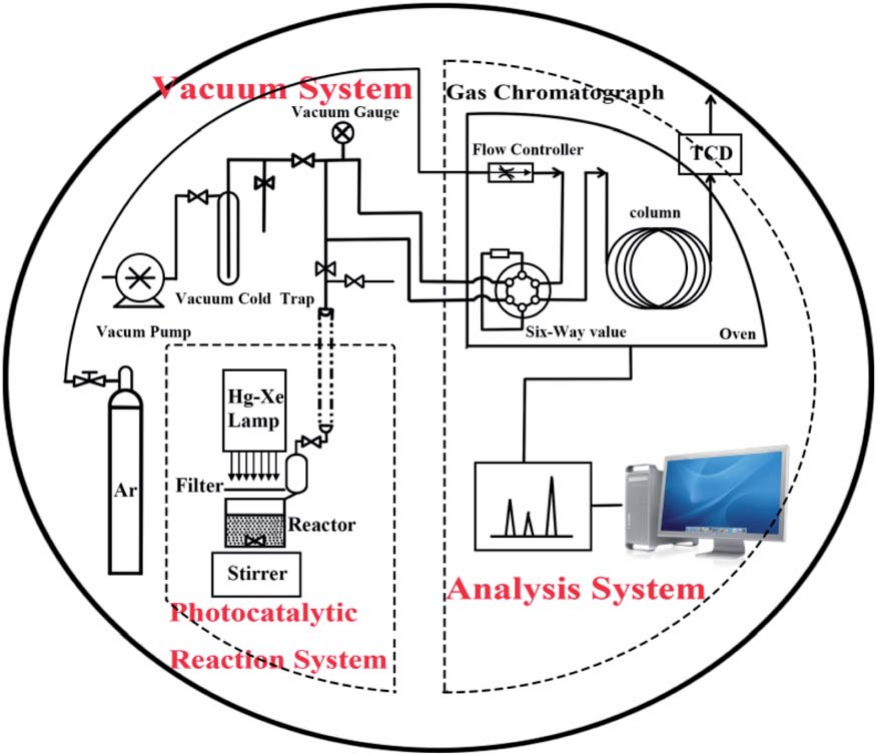
Scheme of photocatalytic reactor and closed gas circulation and evacuation system made of Pyrex glass.

The photocatalytic reactions were carried out in a Pyrex reaction cell connected to a closed gas circulation and evacuation system. Firstly, 50 mg prepared samples and H_2_PtCl_6_ solution (0.004 g mL^−1^) containing 0.5 mg Pt were dispersed in 50 mL of mixed aqueous solution containing 40 mL deionized water and 10 mL sacrificial agent (lactic acid) in a Pyrex reaction cell with constant stirring. Then, the suspension was thoroughly degassed and irradiated by a Xe lamp (300 W) with a cutoff filter (*λ* greater than 420 nm). The amount of H_2_ was analyzed every 30 min using an online gas chromatography. The setup was designed by Liu *et al.*,^23^ as shown in Fig. 1.

## Results and discussion

**Fig. 2:**
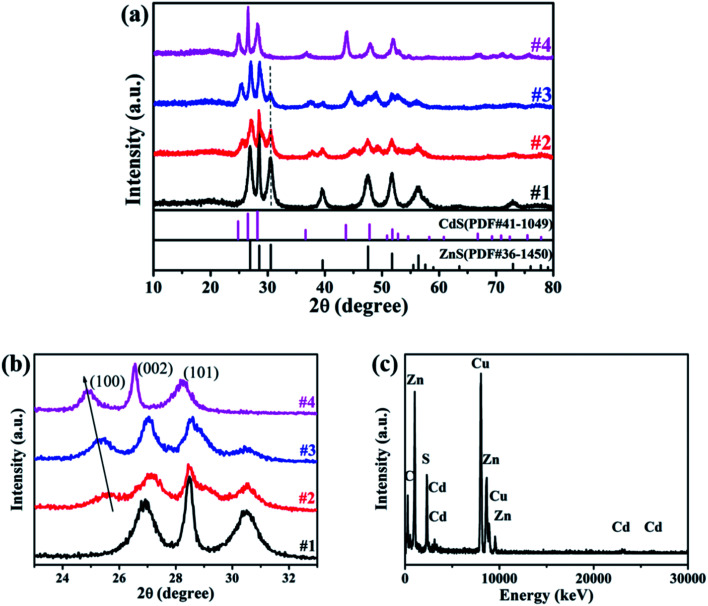
(a) and (b) XRD patterns of samples ZnS (#1), Zn_*x*_Cd_1−*x*_S/ZnS (#2, #3) and CdS (#4); (c) EDX spectrum for sample #3.

The XRD patterns of the prepared samples are shown in Fig. 2a, where the diffraction peaks can be indexed as the hexagonal ZnS (PDF#36-1450) and hexagonal CdS (PDF#41-1049) for samples #1 and #4, respectively. While for samples #2 and #3, the diffraction peaks are composed with both hexagonal ZnS and hexagonal Zn_*x*_Cd_1−*x*_S (PDF#40-0836). With the increase of Cd precursor, the diffraction peaks belonging to Zn_*x*_Cd_1−*x*_S become stronger and the peak positions shift to low-angle side continuously. At the same time, the peak intensity assigned to hexagonal ZnS decreases gradually while its positions is nearly unchanged, indicating that the content of ZnS decreases constantly as shown in the magnified XRD pattern (Fig. 2b). Moreover, from the typical peak positions and Vegard's law, the lattice parameters of Zn_*x*_Cd_1−*x*_S solid solutions varies linearly with composition at constant temperature,^24,25^ from the parameters of sample #2 and #3, we further estimate that *x* is approximately to 0.3 in #2, and 0.2 in #3. The main lattice parameters were displayed in Table 1. The structure and the chemical composition of sample #3 were characterized using EDX spectroscopy, and the EDX spectrum clearly reveals the existence of Zn, Cd and Cu elements in the prepared samples, as shown in Fig. 2c.

**Table 1:** The main lattice parameters for samples #2 and #3

	*d*	(*h k l*)	2*θ*
#2	3.5005	(100)	25.424
	3.2940	(002)	27.047
	3.0912	(101)	28.858
#3	3.5239	(100)	25.252
	3.3105	(002)	26.910
	3.1107	(101)	28.674

The atomic weight percentages of the constituents are given in Table 2, we find that the metal element Zn is dominated in sample #3, and the compositional percentages of (Zn^2+^ + Cd^2+^) and S^2−^ are in the required stoichiometric ratio, but larger than 1. So, there are a lots of sulfur vacancies in the prepared samples.

**Table 2:** Elemental percentage in sample #3 (EDX) data

Element	Atomic percentage %
S	40.15
Zn	54.83
Cd	5.00

**Fig. 3:**
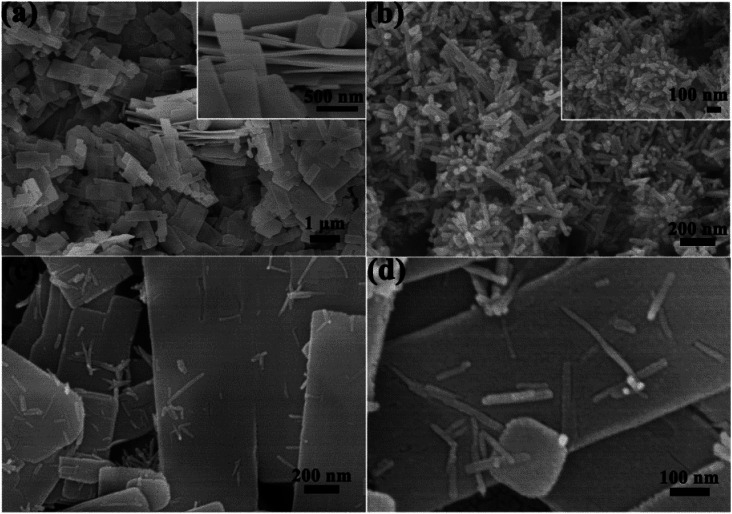
SEM images of samples #1 (a), #4 (b) and #3 (c and d).

The morphologies of samples #1, #3 and #4 were investigated with SEM images as shown in Fig. 3. From Fig. 3a, sample #1 is composed with nanosheets, each nanosheet has a thickness of about 50 nm (inset of Fig. 3a), and sample #4 is composed with nanorods, each individual nanorod has a diameter of several tens nm and length of several hundred nm (Fig. 3b). Low- and high-magnification images of sample #3 were displayed in Fig. 3c and d, which show that after replacing Zn precursor with Cd, the products are mainly composed with nanosheets with some nanorods sparsely growing on the surface of ZnS nanosheets. Sample #2 has the same morphology as sample #3, but it is thicker and narrower than sample #3.

**Fig. 4:**
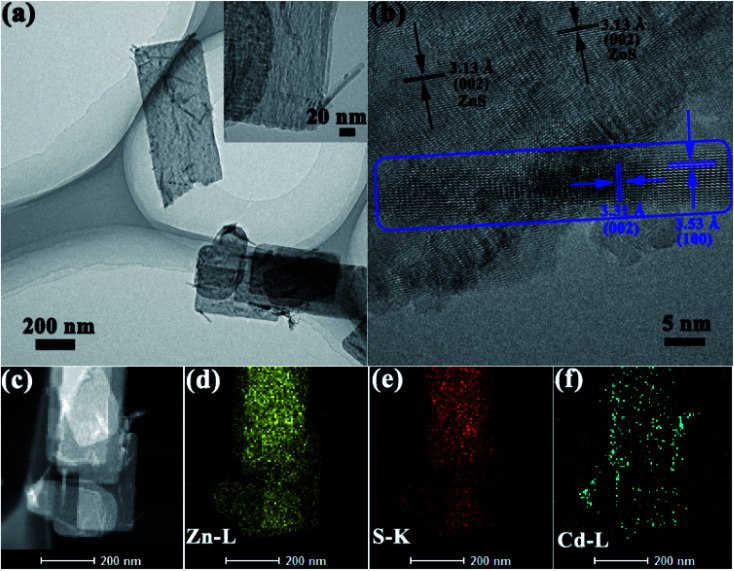
(a) TEM and (b) HRTEM images, (c–f) elemental mappings of sample #3.

TEM and HRTEM measurements were carried out to investigate the microstructure features, and HAADF-STEM to determine the elements distribution. TEM images of selected Zn_*x*_Cd_1−*x*_S/ZnS are displayed in Fig. 4a, the nanorods with a diameter of ∼20 nm were grown on the nanosheets, which is consistent with the result of SEM. Furthermore, from the HRTEM image (Fig. 4b), one can find that the well-resolved lattice fringes with separations of 3.53 and 3.31 Å matched well with the interplanar spacing of (100) and (002) of Zn_0.2_Cd_0.8_S with a high crystallinity, indicating that the Zn_0.2_Cd_0.8_S nanorods grows along the [001] direction. At the same time, the lattice spacing of 3.13 Å in the nanosheet matched well with the (002) plane of hexagonal ZnS. The result is in good agreement with XRD. From elemental mappings in Fig. 4c–f, it is found that Zn and S were well-distributed, while Cd mainly gathered in nanorod area, the results confirm that #2 and #3 were composed of both ZnS nanosheets and Zn_*x*_Cd_1−*x*_S nanorods, and Zn_*x*_Cd_1−*x*_S nanorods were sparsely distributed on the surface of ZnS nanosheets.

Therefore, from the XRD patterns, SEM and TEM images show that samples #2 and #3 were composed with ZnS/Zn_*x*_Cd_1−*x*_S heterostructures with ZnS nanosheets decorated with Zn_*x*_Cd_1−*x*_S nanorods.

**Fig. 5:**
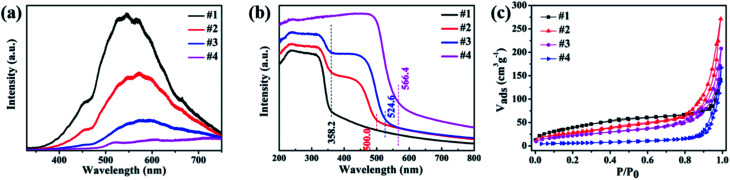
(a) PL spectra, (b) UV-vis absorption spectra and (c) BET surface areas for the prepared samples ZnS (#1), Zn_*x*_Cd_1−*x*_S (#2, #3) and CdS (#4).

The PL spectra were carried out to study the properties of electrons' recombination light emission. As displayed in Fig. 5a, a wide peak from 400 to 700 nm was observed for all the samples, while ZnS (sample #1) has the strongest PL intensity, which was ascribed to the sulfur-related, zinc-related vacancies and the surface states.^26,27^ A decrease in the fluorescence intensity in samples #2 and #3 implies the suppressed recombination of photogenic electron–hole pairs upon attaching Zn_*x*_Cd_1−*x*_S nanorods on ZnS nanosheets, and an increased photogenerated electron–hole separation, and the shift of peak position probably attributed to the transition at the interface.

UV-vis-NIR absorption spectra (Fig. 5b) reveal that samples #1 and #4 have only a single typical absorption edge, while two absorption edges can be easily found in samples #2 and #3, one at ∼358 nm is ascribed to ZnS, the other at ∼500 for sample #2 and ∼525 nm for sample #3 can be attributed to the intrinsic bandgap absorption of Zn_*x*_Cd_1−*x*_S. The observed two absorption edges in samples #2 and #3 reveal the formation of the Zn_*x*_Cd_1−*x*_S/ZnS heterostructures. Sample #3 has a stronger absorption and bigger absorption edge than sample #2. Then, the band gap energy is obtained as 3.46, 2.48, 2.35 and 2.19 eV for samples #1–#4, respectively.

The hollow porous structures of the as-prepared samples were elucidated by nitrogen adsorption/desorption measurements to obtain their BET surface areas. As shown in Fig. 5c, the BET surface area was calculated to be 149.3 m^2^ g^−1^ for pure ZnS nanosheets (#1), and 103.2, 82.3 and 20.9 m^2^ g^−1^ for #2, #3 and #4, respectively. From the results, one can find that pure CdS nanorods possess a low BET surface area, and sample #3 has larger BET surface than sample #2, which is favorable to adsorb molecules.

**Fig. 6:**
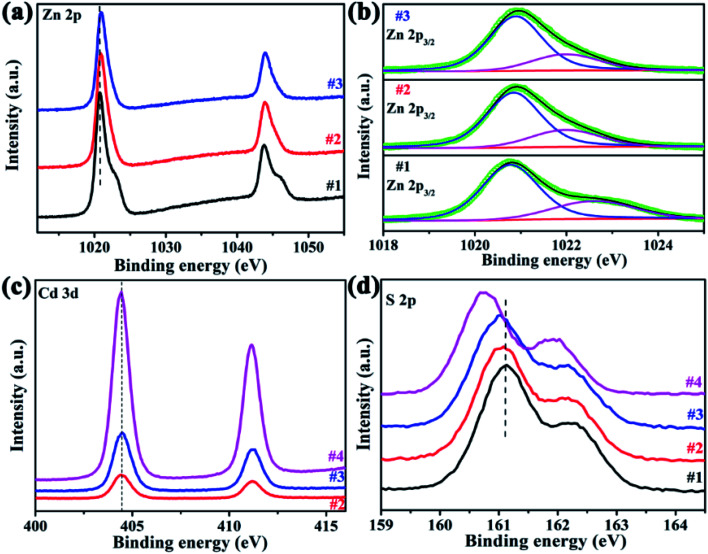
XPS spectra. (a) Zn 2p, (b) deconvolved Zn 2p3_/2_ peaks, (c) Cd 3d and (d) S 2p.

XPS measurements were used to investigate the surface composition of the four samples, the binding energies have been calibrated using the carbon C 1s peak (285.0 eV) as reference. Zn 2p spectra exhibited two peaks at ∼1020.9 eV and 1043.8 eV, which were assigned to Zn 2p_3/2_ and 2p_1/2_ of Zn^2+^, as shown in Fig. 6a. In comparison with sample #1, the Zn 2p binding energy in Zn_*x*_Cd_1-*x*_S/ZnS heterostructures (#2, #3) has a slightly shift to higher energy, indicating the decreased electron density around Zn atoms.^28^ The Zn 2p_3/2_ core level spectra can be deconvolved into two peaks, as shown in Fig. 6b, where the lower one is ascribed to zinc in the lattice, while the higher one was related to the Zn^2+^ in the sulfur deficient regions.^29^ The relative intensity ratios between the higher one and total zinc are equal to 0.298, 0.280 and 0.285 for sample #1, #2 and #3, respectively. That means, many sulfur vacancies exist in sample #2 and #3, and the concentration is approximate, consistent with the EDX results. The Cd 3d XPS spectra are shown in Fig. 6c, in which two peaks at 404.4 eV and 411.1 eV correspond to Cd 3d_5/2_ and 3d_3/2_, respectively, and there is no obvious peak shift between pure CdS nanorods and Zn_*x*_Cd_1-v*x*_S/ZnS heterostructures. For S 2p, the peaks at about 161.0 eV and 162.2 eV are belong to S 2p_3/2_ and S 2p_1/2_, as shown in Fig. 6d. With the introduction of Cd, the peaks of S shift to a lower binding energy, implying that the introduction of Cd atoms leads to the formation of Zn_*x*_Cd_1−*x*_S. The above XPS analyses demonstrated that the electron transformation from Zn and Cd to S in Zn_*x*_Cd_1−*x*_S/ZnS heterostructures can increase electron density of active sites and beneficial to the various reactivity.^30,31^

The apparent quantum efficiency was measured using the 300 W xenon lamp with a 420 nm band pass filters and calculated according to eqn (1):1
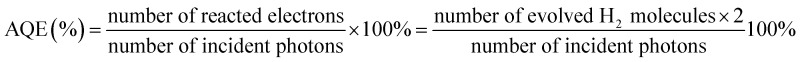


**Fig. 7:**
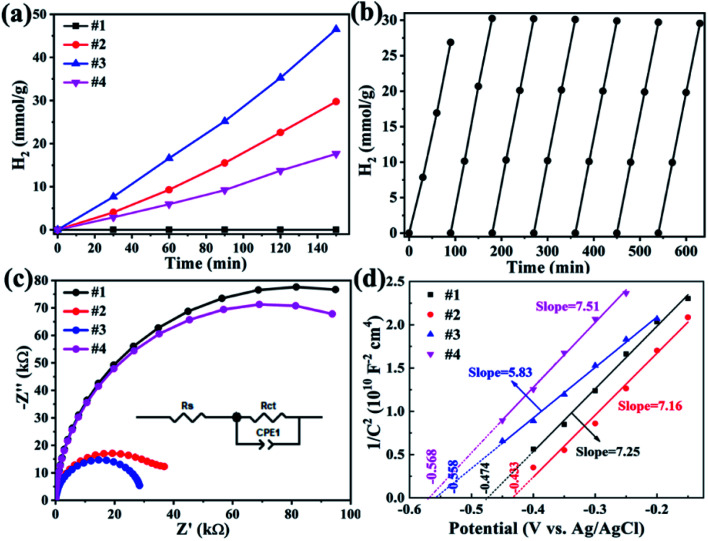
(a)Hydrogen production of pure ZnS CdS and Zn_*x*_Cd_1−*x*_S/ZnS under visible light irradiation (with a 420 nm cut filter), (b) hydrogen production cycle of sample #3; electrochemical performances of all samples, (c) EIS Nyquist plots in a frequency range from 1 to 100 kHz, (d) Mott–Schottky plots at 1000 Hz.

The photocatalytic H_2_ production was carried out under visible light irradiation (with a 420 nm cut filter) for samples ZnS, CdS and Zn_*x*_Cd_1−*x*_S/ZnS, as shown in Fig. 7a. From Fig. 7a, we found that pure ZnS nanosheets exhibit no photocatalytic activity due to the poor visible light absorbability, while ZnS nanosheets decorated with Zn_*x*_Cd_1−*x*_S nanorods reveal an enhanced photocatalytic activity. Sample Zn_0.3_Cd_0.7_S/ZnS has a hydrogen production rate of 9.2 mmol g^−1^ h^−1^, while Zn_0.2_Cd_0.8_S/ZnS (#3) achieved a H_2_ production rate up to 16.7 mmol g^−1^ h^−1^ with an apparent quantum efficiency of 10% at 420 nm, approximately 1.5 times of the former. The value is nearly three times of pure CdS nanorods (5.9 mmol h^−1^ g^−1^).^32^ This result is larger than recently reported 10.8 mmol g^−1^ h^−1^ with Zn_0.5_Cd_0.5_S hybrided with carbon materials as catalysts,^18^ but smaller than the value of 23.264 mmol g^−1^ h^−1^ with Zn_0.5_Cd_0.5_S/dodecahedron ZIF-67 composite.^20^ Moreover, different from the reported severe photocorrosion happened in CdS materials,^33–35^ the as-prepared Zn_0.2_Cd_0.8_S/ZnS heterostructures have a relatively stable H_2_ reproduction with seven consecutive cycles (Fig. 7b), and the initial low rate of hydrogen production was mainly attributed to the activation process of the catalyst.

EIS measurements was carried out to understand the charge transfer process and the electron–hole separation efficiency occurring at the interface between the photoelectrode and electrolyte, as presented in Fig. 7c, the semicircle in the Nyquist plot of each sample corresponds to the parallel combination of *R*_ct_ and CPE1.^36^ The resultant values for the components of the EEC model corresponding to the best fit are presented in Table 3. The smaller diameter corresponds to the lower charge transfer resistance. One can find that sample #3 has smaller charge transfer resistance.

**Table 3:** The EEC impedance parameters

Sample	#4	#1	#2	#3
*R*_s_ (kΩ)	22.31	25.32	25.83	23.16
*R*_ct_ (MΩ)	150.8	164.6	38.73	30.50

Mott–Schottky (M–S) plots were explored to determine the flatband potential (*V*_fb_) for the four sample, as depicted in Fig. 7d. All curves show positive slopes, corroborating the n-type characteristics. M–S plots can also be used to estimate the electron carrier concentration using the following equation.^37^2
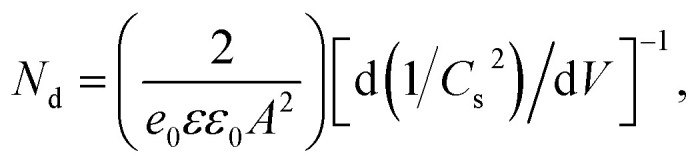
here *e*_0_, *ε* and *ε*_0_ are the electron charge with a value of 1.6 × 10^19^ C, dielectric constant with a value of 10,^38^ and permittivity of vacuum with a value of 8.85 × 10^−12^ F m^−1^, respectively. *A*, *V* and *C*_s_ are the area of the film electrode, the bias applied on the electrode and surface capacitance, respectively. From Fig. 7d, Zn_0.2_Cd_0.8_S/ZnS (#3) has the smallest value of slope, so it has the highest carrier concentration up to 2.42 × 10^19^, larger than the value of 1.95 × 10^19^, 1.98 × 10^19^ and 1.88 × 10^19^ cm^−3^ for sample #1, #2 and #4, respectively. The higher carrier concentration results from the sulfur vacancy. Therefore, the enhanced H_2_ production rate of sample #3 can be ascribed to the improved charge transfer activity. Besides, the flat-band potentials derived from the M–S plots are equal to −0.474, −0.433, −0.558 and −0.568 V *versus* Ag/AgCl electrode at neutral solution for #1, #2, #3 and #4, respectively, that correspond to the related conduction band *versus* Ag/AgCl electrode.^39^

**Fig. 8:**
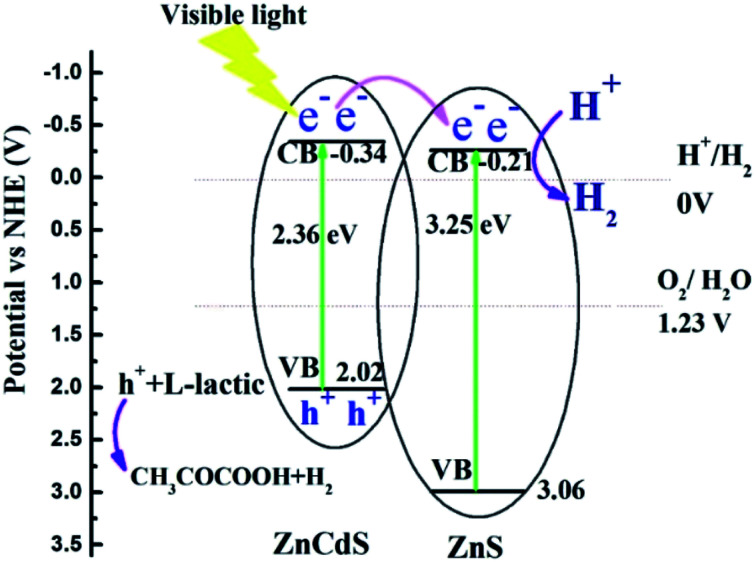
The schematic illustration for electron charge transfer and H_2_ evolution mechanism for the Zn_0.2_Cd_0.8_S/ZnS nanocomposites.

In consideration of band gap energy and the conduction band potential *versus* Ag/AgCl electrode, the energy band alignment can be obtained for sample #3, as shown in Fig. 8. The figure shows that it is type-II heterojunction for Zn_0.2_Cd_0.8_S/ZnS heterostructure. For Zn_0.2_Cd_0.8_S/ZnS heterostructure, under visible-light irradiation (*λ* > 420 nm), electrons will be excited from the conduction band of ZnCdS nanorods to the valence band due to the lower band gap energy. As the conduction potential of ZnCdS is more negative than that of ZnS, the excited electrons will transit from the conduction band of ZnCdS nanorods to that of ZnS nanosheets, resulting in the separation of electron–hole pair. Due to the existence of internal fields, the potential barrier at the interface of the heterostructure prevents photogenerated back to ZnCdS nanorods, the photogenerated electrons have a longer lifetime. At the same time, sample #3 has higher electron carriers, meaning that the electrons could be rapidly transmitted to start reduction reaction with H_2_O, producing hydrogen, while the hole sacrificial agent l-lactic acid in the system consumed electrons, forming pyruvic acid and hydrogen (h^+^ + l-lactic → CH_3_COCOOH + H_2_). These phenomena effectively promoted the photoinduced electron–hole separation and resulted in the excellent photocatalytic performance. The better photocatalytic H_2_ reproduction of sample #3 than sample #2 can be ascribed to the lower charge transfer resistance and the stronger optical absorption, the lower charge transfer resistance will accelerate electrons' transition to participate the reduction reaction with H_2_O; and the stronger visible light absorption of sample #3 can provide more photogenerated electrons for hydrogen production (2H^+^ + 2e^−^ → H_2_).

## Conclusion

In summary, pure ZnS nanosheets, CdS nanorods and Zn_*x*_Cd_1−*x*_S/ZnS composites were synthesized *via* simple hydrothermal and annealing reaction methods. Zn_*x*_Cd_1−*x*_S/ZnS heterostructures were composed with ZnS nanosheets decorated with some Zn_*x*_Cd_1−*x*_S nanorods. Type-II Zn_0.2_Cd_0.8_S/ZnS heterostructures have a lower charge transfer resistance, and stronger optical absorption, as well as a well-matched band energy alignment, hence they achieved a H_2_ production rate up to 16.7 mmol g^−1^ h^−1^ under visible light irradiation (*λ* > 420 nm), approximately 1.5 and 3 times higher than that of Zn_0.3_Cd_0.7_S/ZnS (#2) and pure CdS nanorods (5.9 mmol h^−1^ g^−1^) respectively. The strategy for the construction of heterojunctions can provide more perspective for the preparation of photocatalyst in different application field with a simple preparation method.

## Conflicts of interest

The authors declare no competing financial interest.

## Supplementary Material
